# Supplementary material for: Mosquito Olfactory Response Ensemble enables pattern discovery by curating a behavioral and electrophysiological response database
Source: iScience. 2022 Feb 17;25(3):103938. doi: 10.1016/j.isci.2022.103938 (PMC8899409; doi:10.1016/j.isci.2022.103938)
Supplement: Document S1. Figures S1–S6 [file mmc1.pdf]

iScience, Volume *25*

Supplemental information

Mosquito Olfactory Response Ensemble enables pattern discovery by curating a behavioral and electrophysiological response database

Abhishek Gupta, Swikriti S. Singh, Aarush M. Mittal, Pranjul Singh, Shefali Goyal, Karthikeyan R. Kannan, Arjit K. Gupta, and Nitin Gupta

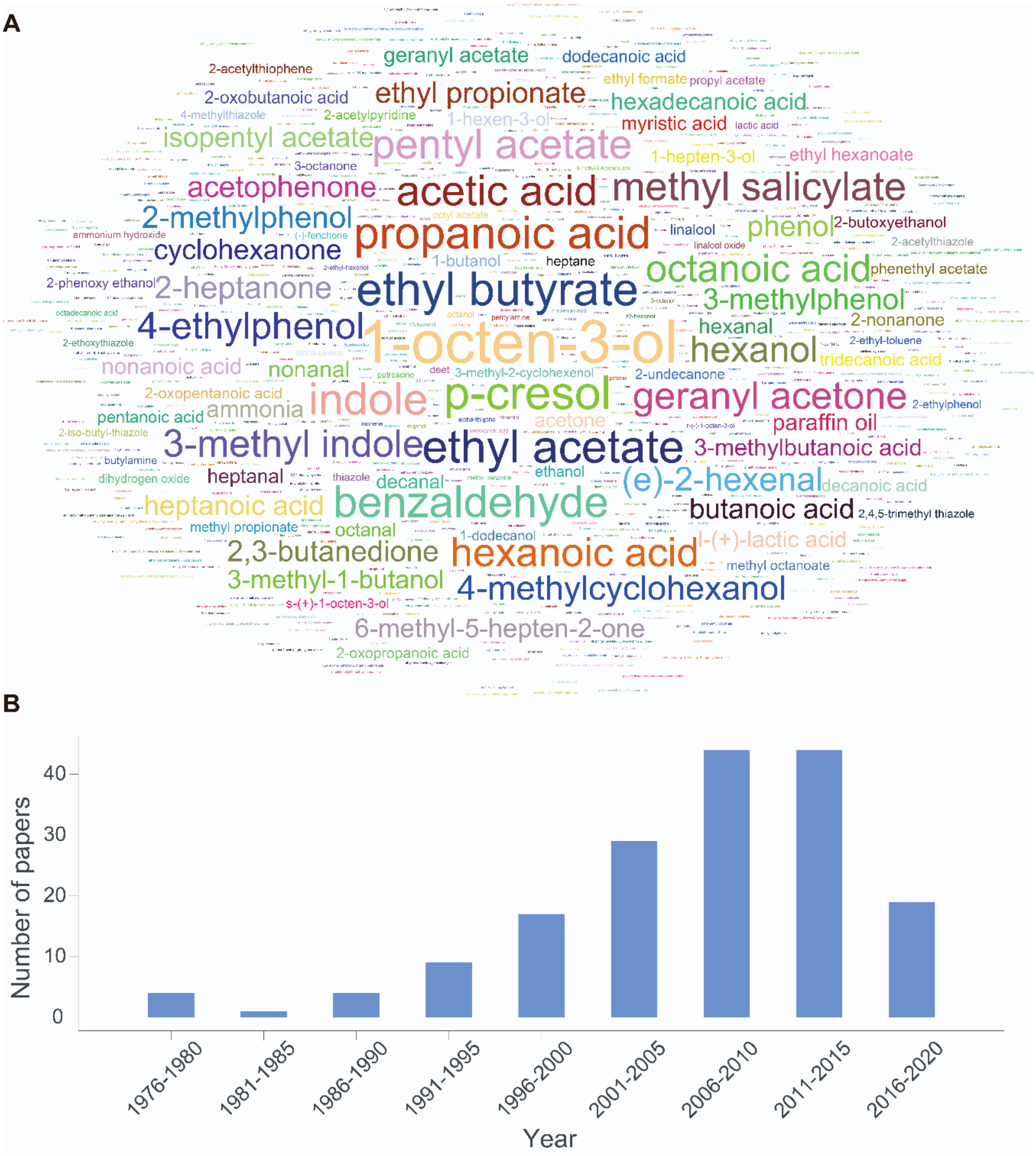

Figure S1. A summary of the dataset, Related to Figure 1.

A, Word cloud showing the odor molecules that are present in the dataset. The size of the text is directly proportional to the number of data-points available for that odor in the dataset. In total, the dataset contains responses for 758 odor molecules. The top 10 odorants with the largest number of data-points (in decreasing order) are: 1-Octen-3-ol, p-Cresol, Ethyl butyrate, Propanoic Acid, Indole, Benzaldehyde, Acetic acid, Pentyl acetate, and Hexanoic acid.

B, Bars indicate the number of studies included from different years (publication date) in the dataset.

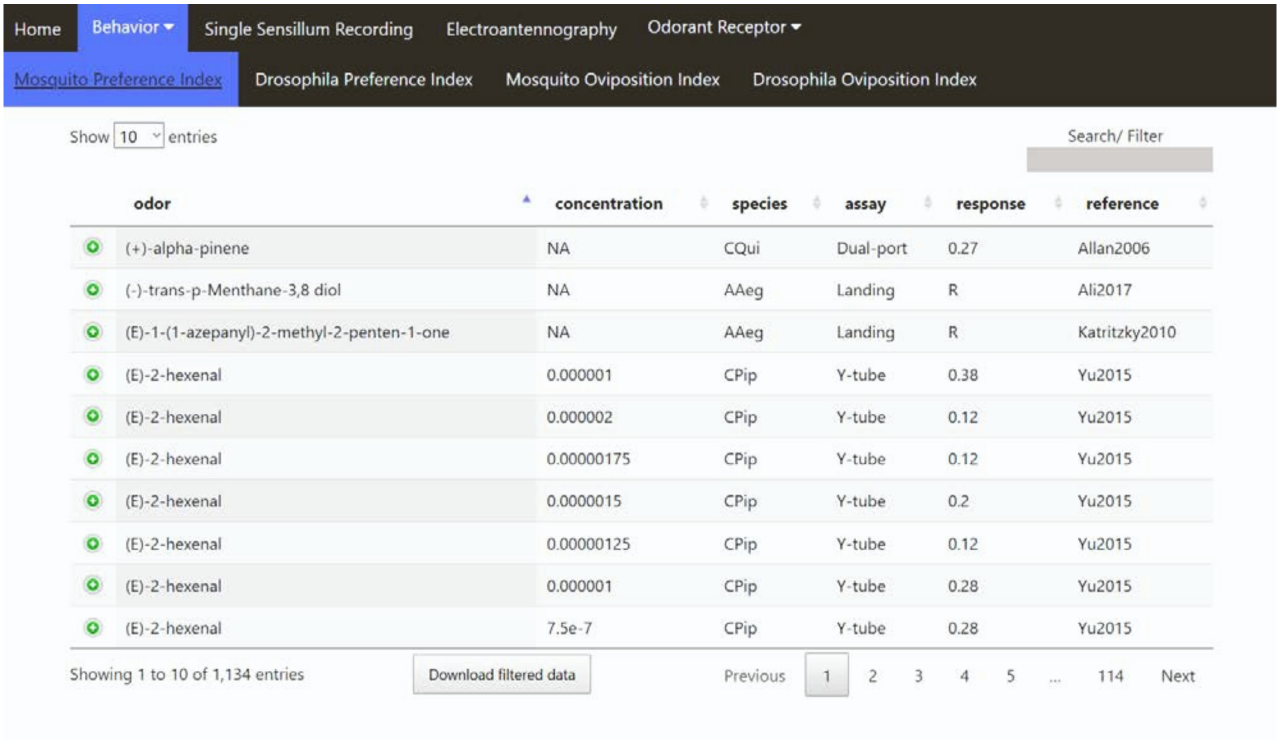

Figure S2. Snapshot of MORE website, Related to Figure 1.

Screeshot shows one of the pages on the MORE website.

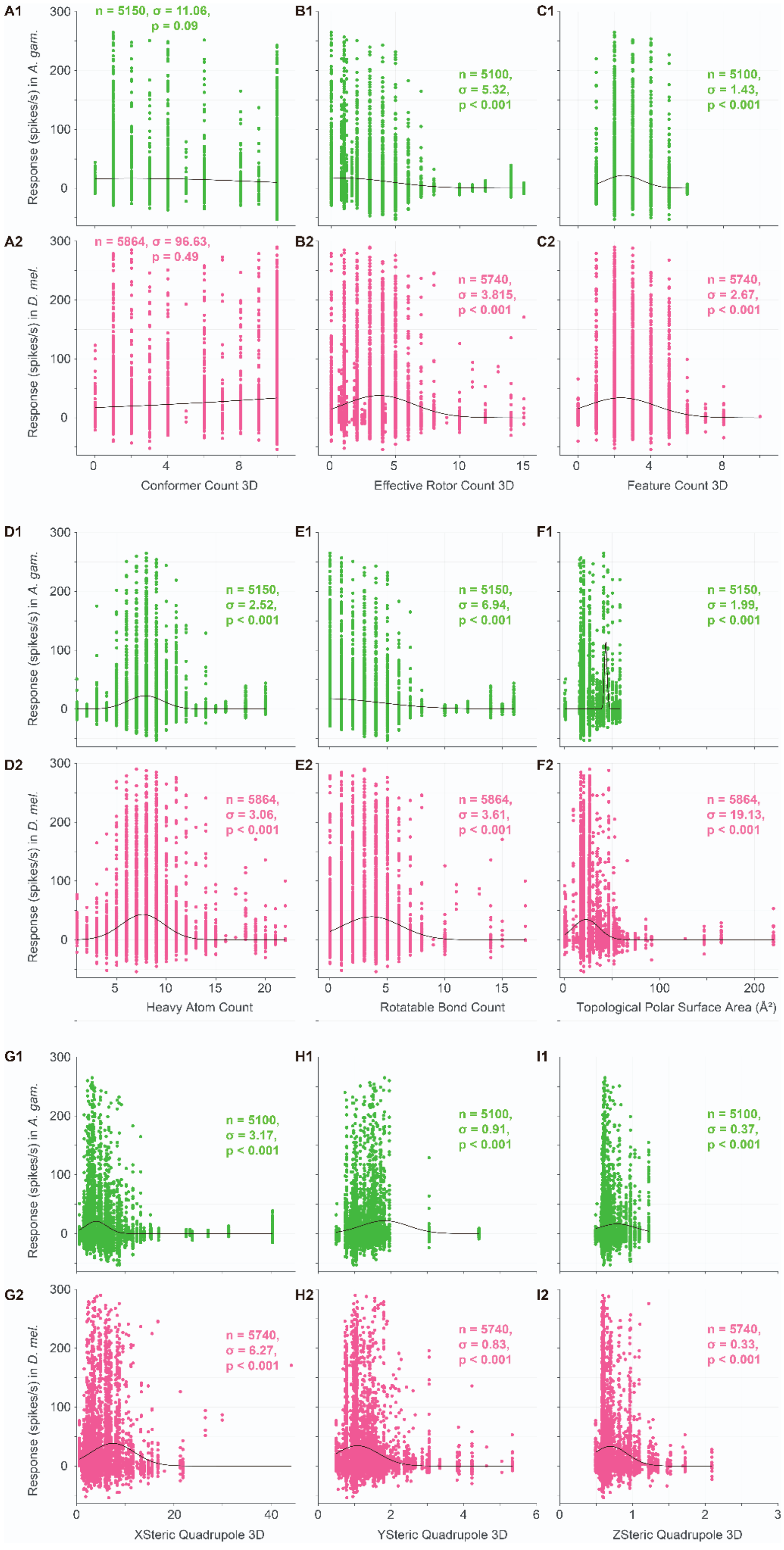
A1
n = 5150, σ = 11.06,
p = 0.09
B1
n = 5100,
σ = 5.32,
p < 0.001
C1
n = 5100,
σ = 1.43,
p < 0.001
A2
n = 5864, σ = 96.63,
p = 0.49
B2
n = 5740,
σ = 3.815,
p < 0.001
C2
n = 5740,
σ = 2.67,
p < 0.001
Response (spikes/s) in A. gam.
Response (spikes/s) in D. mel.
Conformer Count 3D
Effective Rotor Count 3D
Feature Count 3D
D1
n = 5150,
σ = 2.52,
p < 0.001
E1
n = 5150,
σ = 6.94,
p < 0.001
F1
n = 5150,
σ = 1.99,
p < 0.001
D2
n = 5864,
σ = 3.06,
p < 0.001
E2
n = 5864,
σ = 3.61,
p < 0.001
F2
n = 5864,
σ = 19.13,
p < 0.001
Heavy Atom Count
Rotatable Bond Count
Topological Polar Surface Area (Å²)
G1
n = 5100,
σ = 3.17,
p < 0.001
H1
n = 5100,
σ = 0.91,
p < 0.001
I1
n = 5100,
σ = 0.37,
p < 0.001
G2
n = 5740,
σ = 6.27,
p < 0.001
H2
n = 5740,
σ = 0.83,
p < 0.001
I2
n = 5740,
σ = 0.33,
p < 0.001
XSteric Quadrupole 3D
YSteric Quadrupole 3D
ZSteric Quadrupole 3D

Figure S3. Relationship between OR responses and additional physicochemical properties of odor molecules, Related to Figure 2.

Scatter plots of OR responses (in spikes/second) to odors and the physicochemical properties of those odors, including Conformer Count 3D (**A**), Effective Rotor Count 3D (**B**), Feature Count 3D (**C**), Heavy Atom Count (**D**), Rotatable Bond Count (**E**), Topological Polar Surface Area (**F**), XSteric Quadrupole 3D (**G**), YSteric Quadrupole 3D (**H**), ZSteric Quadrupole 3D (**I**) in mosquitoes (**A1**, **B1**, **C1**, **D1, E1, F1, G1, H1, I1**) and *Drosophila* (**A2**, **B2**, **C2**, **D2, E2, F2, G2, H2, I2**). Each point corresponds to an OR-odor pair. In the plots, n is the number of OR-odor pairs, σ is the standard deviation of fitted gaussian, and p represents the p-value.

In all the plots, the black line corresponds to the fitted gaussian.

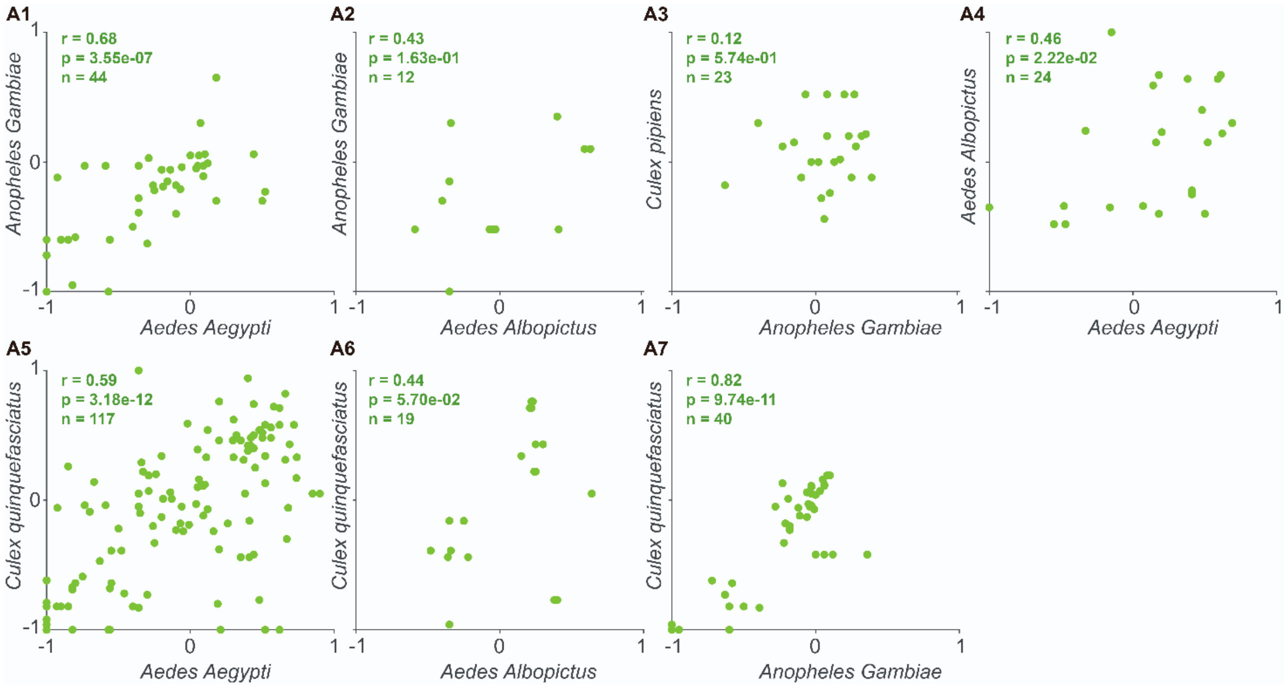

Figure S4. Behavioral preferences across different mosquito species, Related to Figure 5.

Scatter plots of preference indices in pairs of mosquito species.

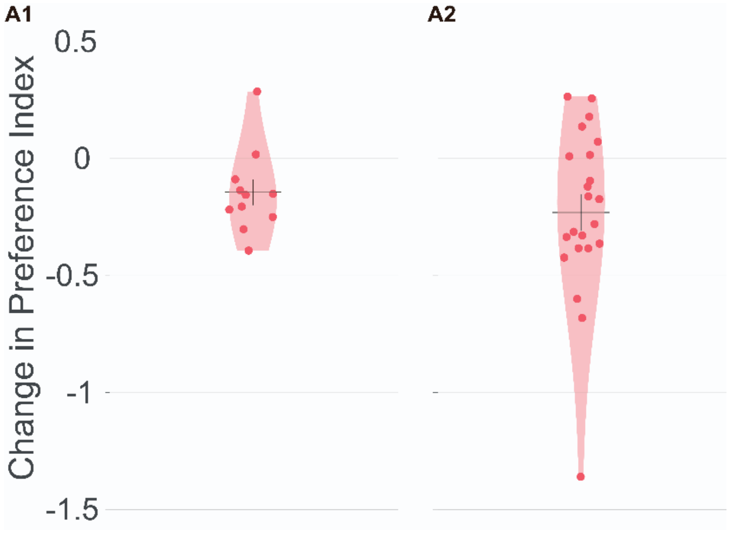

Figure S5. Dependence of behavioral preference on odor concentration, for positive or negative preference indices at the lower concentration, Related to Figure 6.

A1, A2, The change in preference index on increasing the concentration by ten folds when the preference index at the lower concentration is negative (**A1**) or positive (**A2**).

In all plots, the horizontal line and the error bar indicate the mean and the SEM, respectively.

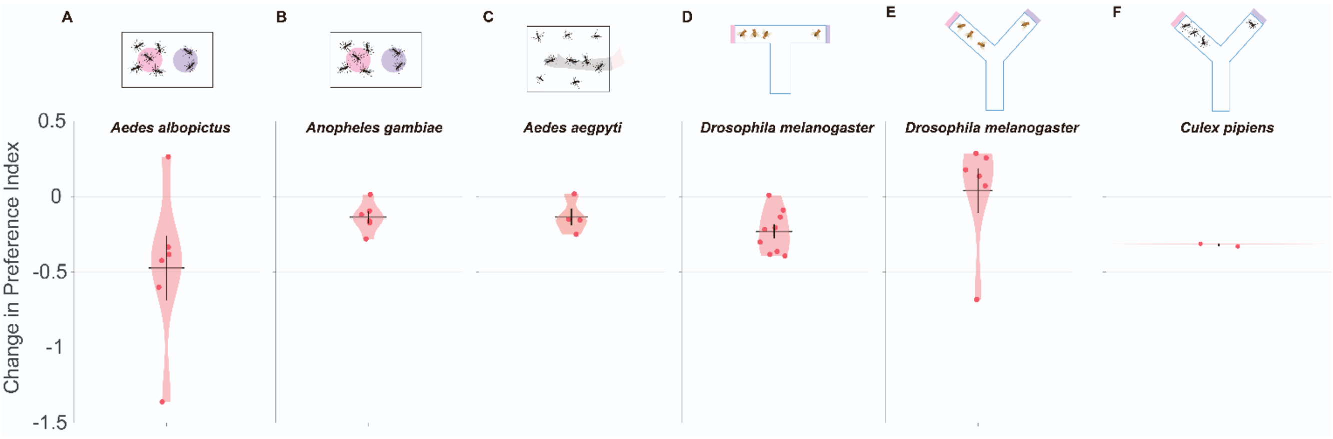

Figure S6. Dependence of behavioral preference on odor concentration for different species, Related to Figure 6.

The change in preference index on increasing the concentration by ten folds for *Aedes albopictus* in dual-port (**A**), *Anopheles gambiae* in dual-port (**B**), *Aedes aegypti* in landing (**C**), *Drosophila melanogaster* in T-maze (**D**), *Drosophila melanogaster* in Y-maze (**E**), and Culex pipiens in Y-maze (**F**) assays.

In all plots, the horizontal line and the error bar indicate the mean and the SEM, respectively.
